# Spurious laboratory results associated with immunoglobulin M gammopathy in a dog with multiple myeloma

**DOI:** 10.1111/jvim.16540

**Published:** 2022-09-20

**Authors:** Samantha C. Loane, Daniel A. Castillo, Anne‐Lorraine D. M. Peschard, Harriet R. Hall, Andre J. Kortum

**Affiliations:** ^1^ Department of Veterinary Medicine University of Cambridge Cambridge Madingley Road United Kingdom

**Keywords:** false‐positive, hypoglycemia, paraprotein, serology

## Abstract

An 11 year old female‐neutered Labrador presented for facial swelling. Clinicopathological abnormalities included hyperglobulinemia, azotemia, hypercalcemia, nonregenerative anemia, thrombocytopenia, and spurious hypoglycemia. Normoglycemia was subsequently confirmed using a cage‐side analyzer (AlphaTRAK, Zoetis, UK). Serum and urine protein electrophoresis documented monoclonal (immunoglobulin M) gammopathy with Bence‐Jones proteinuria. Computed tomography imaging revealed a monostotic osteolytic bone‐lesion, and bone marrow cytology and histopathology documented plasmacytosis with multiple myeloma oncogene 1 / interferon regulatory factor 4 positivity, consistent with multiple myeloma. Infectious disease testing initially indicated seropositivity for *Leishmania*, *Borrelia*, and *Anaplasma* spp.; however, *Leishmania* PCR (splenic and bone marrow aspirates), and paired serological titers for *Borrelia* and *Anaplasma* were negative. Consequently, initial serological results were considered to be false positive because of paraproteinemia‐associated assay interference. Chemotherapy (prednisolone and melphalan combination therapy) was initiated, but the dog was euthanased 30 days later because of the development of pericardial effusion. This is a report of spurious serological (and other laboratory) results occurring secondary to monoclonal gammopathy in a dog.

**Abbreviations**
CT: computed tomography
ELISA: enzyme‐linked immunosorbent assay
HPF: high‐powered field
IFAT: immunofluorescence antibody test
IgM: immunoglobulin M
IRF‐4: interferon regulatory factor 4
MM: multiple myeloma
MUM‐1: multiple myeloma oncogene 1
PEG: polyethylene glycol
POCUS: point of care ultrasound
RI: reference interval
UPC: urine protein : creatinine

## 1 INTRODUCTION

Hyperglobulinemia and monoclonal gammopathy in dogs is most commonly seen secondary to either neoplasia (multiple myeloma, Waldenström's macroglobulinaemia, chronic lymphocytic leukemia, and lymphoma) or infectious diseases (such as ehrlichiosis and leishmaniasis),^1^ and has also been reported secondary to coccidioidomycosis in dogs from endemic regions.^2^ Despite similarities in clinical presentation, treatment and prognosis varies considerably, therefore cases presenting with hyperglobulinemia and monoclonal gammopathy require thorough investigation to ensure a robust diagnosis. Rigorous criteria are used in people to help diagnose multiple myeloma^3^ and are often utilized in veterinary cases, including the presence of abnormal antibodies or antibody fragments (referred to as paraproteins or M‐proteins) in serum or urine (or a combination of both), bone marrow plasmacytosis, and evidence of subsequent organ or tissue impairment (end‐organ damage, including bone lesions).^3^

Circulating paraproteins in human patients cause spurious laboratory results,^4^ including false‐positive serological tests,^5^, ^6^, ^7^, ^8^ pseudohypoglycemia,^9^, ^10^, ^11^ pseudohypophosphatemia,^12^ pseudohyperphosphatemia,^13^, ^14^ pseudohypercalcemia,^15^ falsely‐elevated C‐reactive protein,^16^ and altered bilirubin assays.^17^ Only sporadic case reports exist in the veterinary literature about the effects of paraprotein interference on laboratory tests,^18^, ^19^ and there are no reports documenting false‐positive serological tests in the face of paraproteinemia. This case report describes a dog with multiple myeloma and multiple positive serological tests, postulated to be falsely‐positive secondary to paraproteinemia.

## 2 CASE DESCRIPTION

An 11 year old female neutered Labrador was presented for further investigation of a 48‐hour history of acute onset left‐sided facial swelling. Investigations performed by the referring veterinarian documented thrombocytopenia, anemia, hematuria, and pain when opening the mouth. The dog was prescribed 10 mg/kg paracetamol (Paracetamol; Crescent Pharma Limited) twice daily and 0.1 mg/kg meloxicam (Metacam; Boehringer) once daily before referral. The dog had no pertinent medical history, was up‐to‐date with vaccinations and anthelmintic prophylaxis, and had no history of travel outside the UK.

On presentation, the dog was bright with no evidence of dehydration. Examination documented left‐sided facial swelling, trismus and pain when opening the jaw. An ulcerated mass‐lesion was present at the left oral commissure. Ocular examination documented multifocal retinal hemorrhages, and systolic blood pressure (via Doppler interrogation) was 155 mm Hg, consistent with prehypertension and low risk of target‐organ damage.^20^ No other abnormalities were detected on physical examination.

**FIGURE 1:**
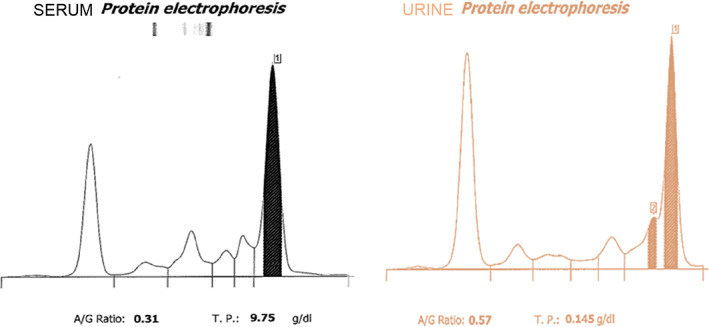
Serum and urine protein electrophoresis documenting IgM monoclonal gammopathy and Bence‐Jones proteinuria

Complete blood count (using a Sysmex XN‐1000 hematology analyzer)^21^ revealed a normocytic normochromic non‐regenerative anemia; hemoglobin concentration 107 g/L (reference interval [RI], 120‐188 g/L), hematocrit 26.7% (RI, 37‐55), reticulocyte count 67.1 × 10^9^/L (RI, 0‐70) with thrombocytopenia 78 × 10^9^/L (RI, 175‐500), and plasma protein above the reference range 110 g/L (RI, 60‐80 g/L). Coagulation profile was within normal limits. Serum biochemistry (using a Beckman Coulter AU840 chemistry analyzer) documented elevated urea 50 mg/dL (RI, 7‐21 mg/dL), creatinine 1.8 mg/dL (RI, 0.4‐1.7 mg/dL), and phosphate 5.7 mg/dL (RI, 2.5‐5.4 mg/dL), which coupled with isosthenuric urine (USG 1.015) was consistent with renal azotemia. It also documented hyperglobulinemia 76 g/L (RI, 24‐47 g/L) with low‐normal albumin 25 g/L (RI, 25‐41 g/L), and hypercalcemia (total) 13 mg/dL (RI, 8.8‐11.6 mg/dL) with normal ionised calcium 5.2 mg/dL (RI, 4.7‐5.6 mg/dL). The most clinically relevant biochemical abnormality was hypoglycemia below the limit of quantification <12.6 mg/dL (RI, 61.3‐101 mg/dL), initially documented via hexokinase assay. However, normoglycemia with an average of 153 mg/dL (lowest reading 139 mg/dL) was subsequently documented using a cage‐side coulometric assay analyzer (AlphaTRAK, Zoetis, UK), suggesting initial analytical error. Electrolytes, hepatobiliary and pancreatic markers, C‐reactive protein, cholesterol, and triglyceride were within normal limits. Urinalysis collected via cystocentesis documented hematuria 150 cells per high‐powered field (hpf) (RI, 0‐5), pyuria 8 cells/hpf (RI, 0‐5), and proteinuria with a urine protein : creatinine (UPC) ratio of 3.52 (RI, 0‐0.4) but no bacteriuria, and negative culture. Serum and urine protein electrophoresis documented a monoclonal immunoglobulin M (IgM) gammopathy and Bence‐jones proteinuria (Figure 1).

Infectious disease testing was performed using cage‐side enzyme‐linked immunosorbent assay (ELISA) kits (SNAP Angio detect, and SNAP 4dx plus, both IDEXX Laboratories Ltd.). These were negative for *Angiostrongylus vasorum*, *Ehrlichia canis/ewingii*, and *Dirofilaria immitis*, but indicated positive antibody detection for *Borrelia burgdorferi* and *Anaplasma phagocytophilum/platys*. Evaluation of acute and convalescent serological titers to *B burgdorferi* and *A phagocytophilum/platys* were subsequently performed but both were negative; (acute and convalescent *B burgdorferi* C6 antibodies <10 ELISA units, RI <10; acute and convalescent *A phagocytophilum/platys* antibodies 0.1 ELISA units, RI <8.0, both IDEXX Laboratories Ltd.), suggesting a previous analytical error. Quantitative ELISA for detection of IgG against *Leishmania* species (IDEXX Laboratories Ltd.) was also positive with a titer of 53.5 enzyme units (RI, <12.0); however, immunofluorescence antibody test (IFAT) serology was inconclusive and subsequent *Leishmania* PCR on splenic and bone marrow aspirates were negative.

Abdominal ultrasonography was performed under sedation. The hepatic parenchyma contained a small rounded hypoechoic nodule (0.89 cm) but was otherwise normal size and shape. There were multiple small rounded hypoechoic nodules throughout the splenic parenchyma, but no other abnormalities were detected on the remainder of the abdominal ultrasound examination. Ultrasound‐guided fine needle aspiration of the liver and spleen documented vacuolar hepatopathy, and high numbers of well‐differentiated plasma cells, respectively.

**FIGURE 2:**
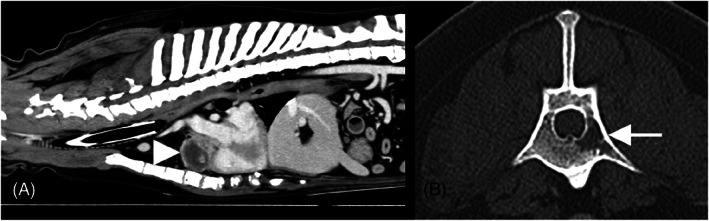
CT images (A: contrast‐enhanced soft tissue window, width 350, level 40, slice thickness 1.00 mm; B: contrast enhanced bone window, width 1500, level 300, slice thickness 1.00 mm) showing the large, rounded heterogeneously contrast‐enhancing heart‐base mass (arrowhead), and a close‐up transverse image of the first lumbar vertebra (B) that was partially included in the thoracic scan, with a left‐sided intra‐medullary ill‐defined osteolytic lesion within the body and left transverse process (arrow)

A contrast‐enhanced computerized tomographic (CT) scan of the head, neck, and chest was performed under general anesthesia. This identified a small, ill‐defined, rounded, poorly contrast‐enhancing swelling on the internal aspect of the left cheek. There was no evidence of local invasion or regional lymphadenopathy, and the lesion did not extend to the ipsilateral tonsil. Thoracic CT identified a large well‐defined heart‐base mass (length 3.8 cm, width 4.8 cm, height 3.97 cm), located ventral to the aortic arch and brachiocephalic trunk, within the pericardium (Figure 2A). Although this mass was mainly of soft‐tissue attenuation with contrast‐enhancement, it also contained several fluid‐filled cavities. No compression of the surrounding vascular structures was evident. An additional finding was the presence of a well‐defined hypoattenuating lesion within the medullary cavity of the body of L1 extending into the left transverse process (Figure 2B), consistent with a monostotic osteolytic bone lesion. The locoregional lymph nodes were within normal limits, with no evidence of pulmonary metastasis. Cytology and histopathology of the oral mass‐lesion indicated neutrophilic stomatitis containing plant material.

Echocardiography documented a large, well‐circumscribed heart‐base mass of heterogeneous echogenicity, located between the aorta and the right atrium within the pericardial space, with no evidence of invasion of the surrounding structures. The heart was otherwise structurally and functionally normal.

**FIGURE 3:**
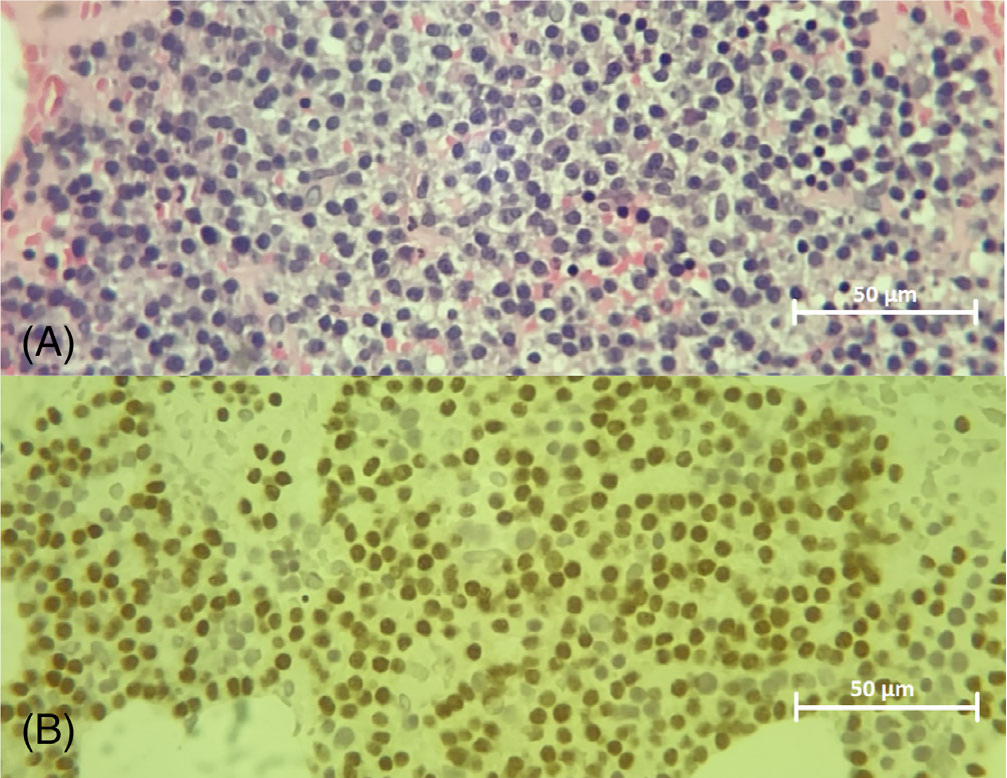
Bone marrow: (A: Histopathology—40× magnification, hematoxylin and eosin stain), documenting markedly increased numbers of well‐differentiated plasma cells frequently forming sheets (64% of the total nucleated cell count) with myelophthisis secondary to plasma cell proliferation (B: Immunohistochemistry—40× magnification, immunolabeling for multiple myeloma oncogene 1 /interferon regulatory factor 4 [MUM1/IRF‐4]). Neoplastic cells exhibit strong nuclear immunolabeling

Bone marrow cytology and histopathology (Figure 3A) documented plasmacytosis (comprising 64% of the total nucleated cell count) with multiple‐myeloma oncogene 1 (MUM‐1) / interferon regulatory factor 4 (IRF‐4) positivity on immunohistochemistry (Figure 3B), and myelophthisis secondary to plasma cell proliferation. In light of the bone marrow plasmacytosis, osteolysis, and the presence of serum and urine paraproteins (monoclonal IgM gammopathy and Bence‐Jones proteinuria), the dog was diagnosed with multiple myeloma.^22^ Given the lack of travel history, low incidence of vector‐borne disease in the UK, and negative subsequent infectious disease testing, concurrent borreliosis, anaplasmosis, or leishmaniasis was considered unlikely and false‐positive results secondary to paraprotein interference were suspected. The neutrophilic stomatitis was considered most likely secondary to multiple myeloma and the increased susceptibility to infection commonly seen in these animals.^23^ The heart‐base mass was considered most likely an unrelated comorbidity, and further investigation of this was declined by the owner because of procedure‐associated risks.

The neutrophilic stomatitis was treated with 20 mg/kg potentiated amoxicillin (Clavaseptin, Vetoquinol UK Limited) twice daily whilst awaiting test results, which resulted in resolution of the left‐sided facial swelling and associated discomfort. After obtaining a diagnosis of multiple myeloma, chemotherapy was initiated with 0.1 mg/kg melphalan (Melphalan, Aspen Pharma) once daily and 0.5 mg/kg prednisolone (Prednisolone, Millpledge Veterinary) once daily, with 1 mg/kg alendronic acid (Alendronate, Accord) once daily because of concurrent hypercalcemia.

There was clinical and biochemical improvement after initiation of treatment, with resolution of the facial swelling and reduction in serum globulin concentration to 57 g/L (RI, 24‐47) after 10 days. The dog represented 4 weeks later with a 24‐hour history of progressive exercise intolerance, tachycardia, and pulse deficits. A thoracic Point‐Of‐Care Ultrasound (POCUS) was performed, identifying a large volume of anechoic pericardial effusion causing collapse of the right atrium and tamponade. The heart‐base mass was thought to be grossly unchanged from the previous thoracic ultrasound. Given the clinical deterioration, the owners opted not to pursue further investigation and elected for euthanasia. A post‐mortem examination was offered but declined.

## 3 DISCUSSION

The pitfalls of paraproteinemia‐induced spurious laboratory results are well known and recognized in human medicine, leading to clinically impossible results,^24^ erroneous diagnoses, unnecessary testing, and incorrect therapies.^25^ Although the exact incidence of paraproteinemia‐induced spurious laboratory results is unknown, up to 35% of samples can be affected.^26^ These are often analytical errors, defined as the effect of a substance present in the sample that alters the correct value of the result because of precipitation, inappropriate binding to assay reagents, or high viscosity and increased turbidity.^4^ In the described case, routine serum biochemistry analysis on the Beckman Coulter AU840 chemistry analyzer initially documented hypoglycemia below the limit of quantification (<12.6 mg/dL) despite repeated assessment on a cage‐side analyzer confirming normoglycemia, with no applicable clinical signs. This is likely attributable to the difference in assay methodology. The former analyses glucose via the hexokinase method, which is the preferred method for glucose analysis. Paraprotein interference can occur because of antibody cross‐reactivity against antigens involved in the hexokinase reaction,^9^, ^10^, ^11^, ^24^, ^27^ or increased precipitation and turbidity^27^ impacting on the optical change in the assay. This is common in people, particularly with IgM, because of its high molecular weight and ability to form polymers, thus reducing its solubility.^4^ Conversely, cage‐side glucose monitors (such as the AlphaTRAK glucometer) measure glucose via a coulometric electrochemical sensor, which is a dry chemistry method allowing measurement of the analyte concentration in a protein‐free filtrate,^9^ and is therefore unaffected by paraproteinemia.

Along with spurious chemistry results, false‐positive serological results occur in humans secondary to paraproteinemia, both because of hematological malignancies (such as multiple myeloma) and secondary to benign age‐related increases in immunoglobulin levels. This includes false‐positive Galactomann antigen tests for aspergillosis,^8^, ^28^, ^29^ false‐positive serological tests for syphillis,^5^, ^29^, ^30^ and false‐positive rK39 rapid diagnostic tests for leishmaniasis.^6^ There are also false‐positive results for a wide range of infectious agents (viral, bacterial, and protozoal) in human patients receiving immunoglobulin therapy IV.^31^, ^32^, ^33^, ^34^, ^35^ The mode of interference in the *Leishmania* assay in the described case could be associated with the paraprotein cross‐linking the antigen with the conjugate antibody,^28^ or an inability to remove unbound antibody conjugate by the washing procedure. Alternatively, increased turbidity and precipitation could have affected measurement of the optical density and absorbance. Determining the exact mechanism is complex and would require further studies. Given that veterinary serological testing often relies on similar ELISA technology, paraprotein interferences could plausibly occur in animals secondary to paraproteinemia. Clinicians should be aware of these potential analytical errors as many clinical and clinicopathological abnormalities (anemia, thrombocytopenia, hyperglobulinemia) occur secondary to both infectious etiologies and multiple myeloma, and serological testing is therefore commonly performed in the investigation of these cases.

In this case, although there was no history of foreign travel, infectious disease was still initially considered a possible etiology, given that anaplasmosis,^36^ ehrlichiosis,^37^ and leishmaniasis^38^ have been previously reported to occasionally occur in UK dogs without a history of foreign travel. Initial positive serological results for *A phagocytophilum/platys*, *B burgdorferi*, and *Leishmania* spp. therefore prompted further evaluation using confirmatory tests. There is no gold standard diagnostic test for leishmaniasis.^39^ Molecular methods such as PCR are highly sensitive for organism detection, but sensitivity is determined by the sites sampled. A positive PCR result or direct visualization of amastigotes (especially from splenic or bone marrow aspirates) is generally considered conclusive for infection. Serology is regarded as a good screening test for clinically affected animals,^39^ and of the serological tests, IFAT has the highest sensitivity^39^ for clinical disease and was equivocal in this case. Given the lack of a high IFAT titer in combination with negative PCR results and lack of travel history outside the UK, it was considered unlikely that the dog was infected with *Leishmania* spp.

Although the SNAP 4dx plus test (IDEXX Laboratories Ltd) is highly sensitive, false‐positives can also occur, particularly in regions with low disease prevalence.^40^, ^41^ Given that both borreliosis and anaplasmosis are uncommon in the UK, and considering the other diagnostic findings in this case, paired serology was therefore performed to provide further evidence of infection status. The negative acute and convalescent titers ruled out infection and, given the concurrent paraproteinemia and other spurious laboratory abnormalities, previous false‐positive results because of paraprotein interference were suspected.

Because of the known effect of paraproteinemia causing spurious laboratory results in human medicine, multiple protocols have been developed to try and address this and improve test accuracy. Some authors propose serial dilution of samples with saline until concordant results are achieved,^42^ but dilution with saline might not yield reportable results because it is difficult to predict how clonal immunoglobulins will behave in vivo and at particular concentrations.^13^ Serial dilution can diminish or eliminate disturbances caused by interference with sample constituents, but if the underlying etiology of spurious laboratory results is because of paraprotein precipitation it might still not be possible to obtain an unbiased result. In this case, sample dilution with an equal volume of deionized water avoided paraprotein interference of glucose measured by the hexokinase method.

An alternative method to avoid interference includes deproteination of the sample.^27^ Polyethylene glycol (PEG) is a polymer of ethylene glycol that precipitates proteins by steric hindrance without denaturing or interfering with them.^13^ Protocols have been published describing PEG precipitation to minimize or reduce paraprotein interference,^43^ and studies have assessed PEG precipitation for multiple chemistry analytes, including creatinine^44^ and phosphate^13^ in human medicine, and could also be considered in veterinary medicine. Use of a different analytical method (eg, dry chemistry) could also be used, which eliminates the effects of large molecules such as paraproteins.

A limitation in this case was the lack of cytological or histopathological evaluation of the heart‐base mass, and as such we cannot conclude the underlying etiology of this lesion and whether it was an incidental comorbidity in an older animal (such as chemodectoma), or a rare presentation of extramedullary plasmacytoma. Mediastinal plasmacytoma occurring in people with multiple myeloma is very rarely documented, with only 20 cases in the literature,^45^ and was thus considered unlikely in this case. Another limitation is the short follow‐up time because of euthanasia, thus precluding follow‐up infectious disease testing in the face of normalized globulin concentrations.

## CONFLICT OF INTEREST

The authors declare no conflict of interest.

## OFF‐LABEL ANTIMICROBIAL DECLARATION

The authors declare no off‐label use of antimicrobials.

## INSTITUTIONAL ANIMAL CARE AND USE COMMITTEE (IACUC) OR OTHER APPROVAL DECLARATION

Written owner consent was obtained at the time of animal admission.

## HUMAN ETHICS APPROVAL DECLARATION

The authors declare human ethics approval was not needed for this study.
